# A practical approach to in-hospital management of new-onset refractory status epilepticus/febrile infection related epilepsy syndrome

**DOI:** 10.3389/fneur.2023.1150496

**Published:** 2023-05-12

**Authors:** Zubeda Sheikh, Lawrence J. Hirsch

**Affiliations:** ^1^Department of Neurology, West Virginia University School of Medicine, Morgantown, WV, United States; ^2^Epilepsy Division, Department of Neurology, Yale School of Medicine, New Haven, CT, United States

**Keywords:** new-onset refractory status epilepticus, febrile infection related epilepsy syndrome, anakinra, tocilizumab, rituximab, super-refractory status epilepticus, neuroinflammation, autoimmune encephalitis

## Abstract

New-onset refractory status epilepticus (NORSE) is “a clinical presentation, not a specific diagnosis, in a patient without active epilepsy or other preexisting relevant neurological disorder, with new onset of refractory status epilepticus without a clear acute or active structural, toxic, or metabolic cause.” Febrile infection related epilepsy syndrome (FIRES) is “a subcategory of NORSE that requires a prior febrile infection, with fever starting between 2 weeks and 24 h before the onset of refractory status epilepticus, with or without fever at the onset of status epilepticus.” These apply to all ages. Extensive testing of blood and CSF for infectious, rheumatologic, and metabolic conditions, neuroimaging, EEG, autoimmune/paraneoplastic antibody evaluations, malignancy screen, genetic testing, and CSF metagenomics may reveal the etiology in some patients, while a significant proportion of patients’ disease remains unexplained, known as NORSE of unknown etiology or cryptogenic NORSE. Seizures are refractory and usually super-refractory (i.e., persist despite 24 h of anesthesia), requiring a prolonged intensive care unit stay, often (but not always) with fair to poor outcomes. Management of seizures in the initial 24–48 h should be like any case of refractory status epilepticus. However, based on the published consensus recommendations, the first-line immunotherapy should begin within 72 h using steroids, intravenous immunoglobulins, or plasmapheresis. If there is no improvement, the ketogenic diet and second-line immunotherapy should start within seven days. Rituximab is recommended as the second-line treatment if there is a strong suggestion or proof of an antibody-mediated disease, while anakinra or tocilizumab are recommended for cryptogenic cases. Intensive motor and cognitive rehab are usually necessary after a prolonged hospital stay. Many patients will have pharmacoresistant epilepsy at discharge, and some may need continued immunologic treatments and an epilepsy surgery evaluation. Extensive research is in progress now via multinational consortia relating to the specific type(s) of inflammation involved, whether age and prior febrile illness affect this, and whether measuring and following serum and/or CSF cytokines can help determine the best treatment.

## Introduction

Status epilepticus (SE) is a neurologic emergency. A third of patients fail to respond to benzodiazepines and one other anti-seizure medication (ASM) and are, therefore, by definition (failing two ASMs), classified as having refractory status epilepticus (RSE) (1, 2). Attempts at seizure control are accompanied by simultaneous evaluation for the underlying etiology, the targeted treatment of which is essential to stop the seizures. In a significant minority of patients, an extensive diagnostic workup fails to reveal the cause of SE. This group represents two-thirds of *de novo* refractory status epilepticus (3).

A multinational panel of experts defined new-onset refractory status epilepticus (NORSE) as “a clinical presentation, not a specific diagnosis, in a patient without active epilepsy or other preexisting relevant neurological disorder, with new onset of refractory status epilepticus without a clear acute or active structural, toxic, or metabolic cause. This includes patients with viral or autoimmune causes. If no cause is found after extensive evaluation, this is considered ‘cryptogenic NORSE’.” Febrile infection-related epilepsy syndrome (FIRES) is “a subcategory of NORSE that requires a prior febrile infection, with fever starting between 2 weeks and 24 h before the onset of RSE, with or without fever at the onset of SE. This applies to all ages. There may or may not be fever at the onset of SE (4).” Patients with FIRES account for the majority (~90%) of pediatric NORSE (5). The rarity of NORSE and the varied etiologies (when one is identified) have challenged impactful research in understanding the therapeutics. Numerous case reports, series, and reviews have been published (6), but there have been no randomized controlled trials to guide management.

A 2017 survey of neurointensivists showed that two-thirds of responding institutions did not have a protocol for evaluating and managing NORSE, a quarter of respondents would not perform autoimmune work-up, and a third would never use Intravenous immunoglobulins (IVIG) (7). In the absence of direct evidence guiding management and the variability in management practices shown in this survey, standardization of terminology was felt to be an important first step, followed by consensus recommendations for clinical management. Standardized terminology was proposed for NORSE and FIRES at the first International NORSE/FIRES Symposium in 2017 in Salzburg, Austria, conducted before the 6th Colloquium on Status Epilepticus and Acute Seizures, resulting in the definitions above (4). A recent Delphi study attempts to guide management; this was conducted to map the existing literature and multinational, multidisciplinary expert opinion to a list of consensus recommendations for treating NORSE/FIRES in all age groups (8). After a literature review, 48 experts rated the recommendation statements regarding diagnosis, treatment, and research directions on a scale of 1 (strong disagreement) to 9 (strong agreement). The consensus was reached (the statement was appropriate) if it received a median score of ≥7, whereas inappropriate if the median score was three or less. The analysis of evidence was mapped to the results of each statement included in the Delphi study. However, the evidence supporting most recommendations is limited; thus, these are intended to be considerations rather than strict guidelines.

## Methods

Relevant articles from the annotated reference list of over 130 articles on NORSE/FIRES maintained by the NORSE institute were chosen for a detailed review (6). This list was last updated in July 2022 with input from the authors and other members of the NORSE Institute. In addition, Pubmed and Google Scholar searches were performed using the search terms “NORSE,” “FIRES,” “new-onset refractory status epilepticus,” “febrile infection-related epilepsy syndrome,” “refractory status epilepticus,” and “super-refractory status epilepticus” to generate the updated articles for review, including those published after July 2022. Permission was obtained to use the tables listing diagnostic evaluation on the NORSE institute website, and these were revised based on the updated article review. A flowchart was created to show an algorithmic approach to evaluating and managing NORSE/FIRES based on the information obtained from the review of the articles.

## Diagnostic approach

**Figure 1:**
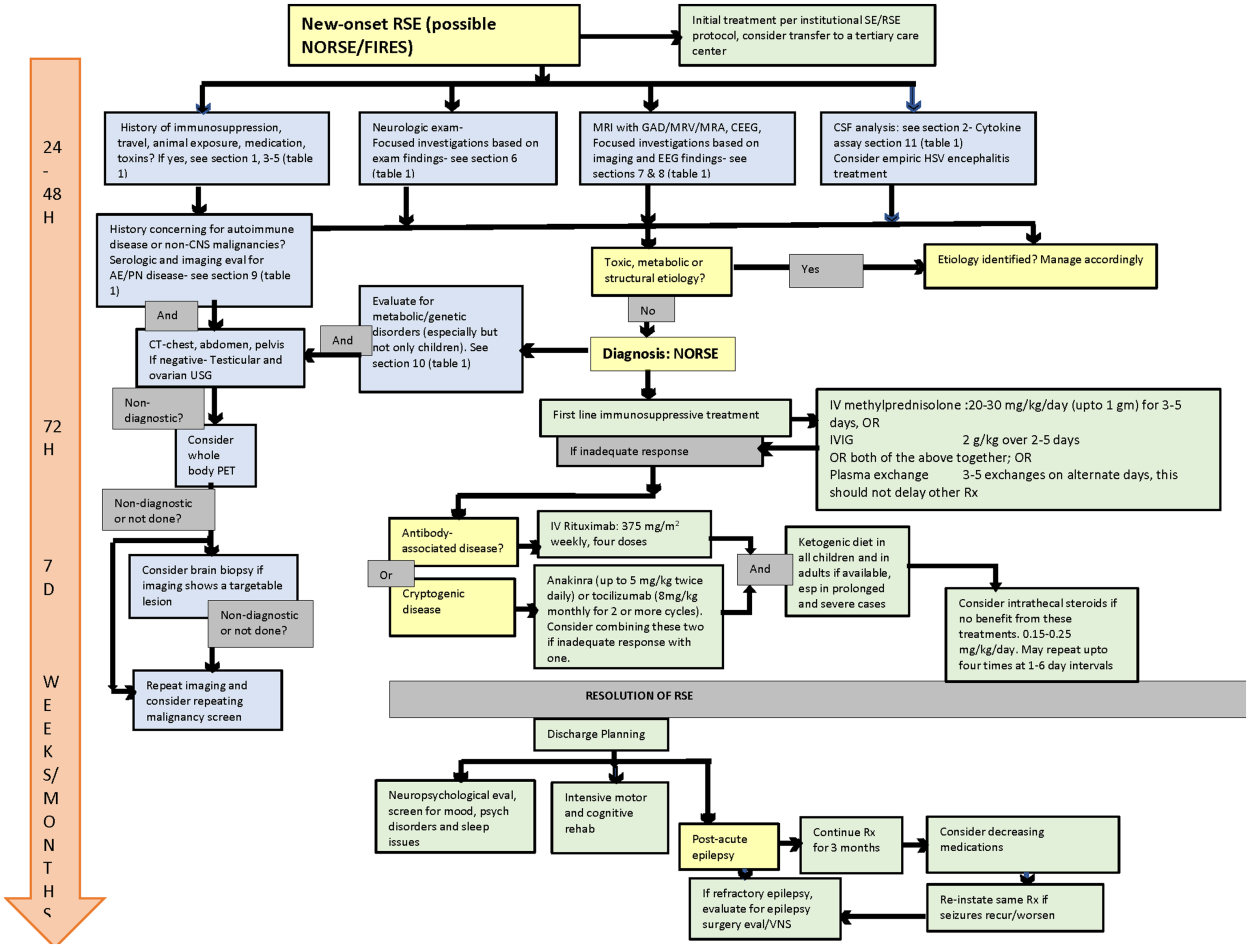
Flow diagram for evaluation and management of NORSE/FIRES. NORSE, new onset refractory status epilepticus; FIRES, febrile infection-related epilepsy syndrome; SE, status epilepticus; RSE, refractory status epilepticus; GAD, gadolinium; MRV, magnetic resonance venogram; MRA, magnetic resonance angiography; CEEG, continuous EEG; USG, ultrasonography; PET, positron emission tomography; HSV, herpes simplex virus; VNS, vagus nerve stimulator. 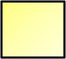 Diagnostic consideration. 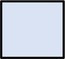 Diagnostic procedure. 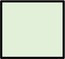 Treatment. 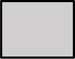 Outcome assessment. 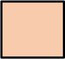 Management timeline.

Acute management of adults with NORSE/FIRES should be primarily directed by neurointensivists when available and in consultation with a multidisciplinary team, including epilepsy, rheumatology, and immunology, at a center with the capability for continuous EEG monitoring (cEEG) and ideally at a tertiary care center with expertise in RSE, including NORSE (8, 9). By the time NORSE is suspected, the initial evaluation, including blood counts, chemistry, liver/renal function parameters, electrolytes, toxicology screen, CNS imaging, and preliminary CSF analysis, have been done and have failed to determine a cause for the RSE. There have been a few papers published suggesting a timed approach to the evaluation and management of NORSE/FIRES (9, 10). In Figure 1, we incorporate the suggestions from these papers to the most recent consensus recommendations obtained via the Delphi methodology and a literature review to create a comprehensive algorithm to guide the diagnosis and management of NORSE/FIRES (8, 9).

### Blood/CSF investigations

Table 1 section 1 lists the tests to consider in the initial evaluation of blood/serum/CSF. Section 3 of Table 1 shows the additional blood/serum tests to consider if, on history, any high-risk features are suspected, such as an immunocompromised state or geographic, seasonal, or occupational exposure. Additional testing may be necessary for specific possible zoonotic exposures (shown in section 4 of Table 1) or exposure to drugs and toxins (section 5). This aligns with the expert consensus to obtain a comprehensive infectious evaluation in all patients, including cultures and viral and bacterial serology relevant to the geographic region and season (sections 1–3) (8, 9). In addition to the above, the expert consensus recommends obtaining the following tests in all or most patients in the initial 48 h (8): Comprehensive rheumatologic evaluation (section 9), evaluation for inborn errors of metabolism in young children (section 10), autoimmune and onconeural antibody panel (section 9), and extra blood and CSF samples for storage for future analysis (e.g., cytokine and genetic analyses) (section 11).

**Table 1:** List of heterogeneous etiologies of NORSE/FIRES and the diagnostic tests to consider (4, 8, 9, 11–26).

*Section 1: Initial metabolic/infectious work up*
**Blood:** CBC, BMP, LFT, BUN, Electrolytes (Ca, Mg, Phos), ESR, CRP, bacterial and fungal cultures **Serum:** RPR-VDRL, HIV-1/2 immunoassay with confirmatory viral load if appropriate, PPD placement, IgG and IgM testing for *Chlamydia pneumoniae*, *Bartonella henselae*, *Mycoplasma pneumonia*, *Coxiella burnetii*, *Shigella* species, and *Chlamydia psittaci* Anti-neuronal surface antibody panel and onconeural antibodies (see below) Cytokines (see below) **Nares:** Respiratory viral DFA panel
*Section 2: CSF studies*
Cell count and differential count, protein, glucose, lactate and pyruvate (ratio lactate/pyruvate). Bacterial and fungal stains and cultures. PCR for HSV1, HSV2, VZV, EBV, HIV, *C. pneumoniae*, *B. henselae*, *C.burnetti*, *C psittaci*, *Shigella* species, VDRL, M Tb PCR. Immunoelectrophoresis/electrofocusing and cytology Anti-neuronal surface antibody panel and onconeural antibodies (see below) Cytokines (see below) Store CSF for metagenomic next-generation sequencing
*Section 3: Focused testing for high-risk features*
**Recommended in immunocompromised patients:** Serologic: IgG *Cryptococcus* species, IgM and IgG *Histoplasma capsulatum*, IgG *Toxoplasma gondii* Sputum: M Tb Gene Xpert (molecular test for tuberculosis) CSF: Eosinophils, silver stain for CNS fungi, PCR for JC virus, CMV, EBV, HHV6, EEE, Enterovirus, Influenza A/B, HIV, WNV, Parvovirus. Listeria Ab, Measles (Rubeola), Toxoplasma IgG Stool: Adenovirus PCR, Enterovirus PCR **Recommended if geographic/seasonal/occupational risk of exposure:** Serum: buffy coat and peripheral smear (for parasitic infections such as malaria, babesiosis, toxoplasmosis etc.), Lyme EIA with IgM and IgG reflex, *Acanthamoeba* spp., *Balamuthia mandrillaris*, *Baylisascaris procyonis* Serum and CSF: samples to CDC DVBID Arbovirus Diagnostic Laboratory, CSF and serum Rickettsial disease panel, Flavivirus panel, Bunyavirus panel Other optional: see attached table for further geographical/zoonotic risk factors
*Section 4: Additional zoonotic/geographic exposure considerations*
**Ingestion:** Unpasteurized milk: Tick-borne virus, *C. burnetii* Star fruit: caramboxin, oxalic acid **Geographical factors:** (residence, recent travel) Africa: West Nile virus Australia: Murray Valley Encephalitis virus, Japanese Encephalitis virus, Hendra virus, Eastern Equine virus, Western Equine virus, Venezuelian Equine virus Central and South America: Saint-Louis virus, *Rickettsia* spp. West Nile virus, Tick-borne virus, *Ehrlichia chaffeensis*/*Anaplasma phagocytophilum* Europe: Japanese virus West Nile virus India, Nepal: Tick-borne virus Middle East, Russia, Southeast Asia, China, Pacific Rim: Japanese virus, Tick-borne virus, Nipah virus **Seasonal factors:** Late summer/early fall or winter: arboviruses, enteroviruses, influenza virus **Animal exposure:** Cats—*B. henselae*, *T. gondii* Horses—Eastern Equine virus, Western Equine virus, Venezuelian Equine virus, Hendra virus Raccoons—*Baylisascaris procyonis* Rodents—*Bartonella Quintana*, Eastern Equine virus, Western Equine virus, Tick-borne virus, Powassan virus, LaCrosse virus Sheep and goats—*C. Burnetii* Swine—Japanese virus, Nipah virus **Insect exposure, including travel to infested area:** Mosquitoes: EEE, WEE, Venezuelan Equine virus, Saint-Louis virus, Murray Valley virus, Japanese virus, West Nile virus, La Crosse virus Tick-borne virus, Powassan virus, *Rickettsia* spp. Ticks: *E. Chaffeensis*/*A. Phagocytophilum*
*Section 5: Status epilepticus caused by drugs, toxins, or related to medical intervention*
**Drugs:** Antibiotics: cephalosporins, carbapenems, quinolones isoniazid, mefloquine, chloroquine Antidepressants/antipsychotics: bupropion, tricyclic antidepressants especially amoxapine, selective serotonin reuptake inhibitors, venlafaxine, lithium Chemotherapy: platinum-based agents cytarabine, gemcitabine irinotecan interferon-alpha, interleukin-2 Humanized monoclonal antibodies: bevacizumab, ipilimumab, rituximab, infliximab Tyrosine kinase inhibitors: imatinib, pazopanib, sorafenib, sunitinib, GMCSF, ifosfamide Immunosuppressive and immunomodulatory drugs: cyclosporine, tacrolimus, sirolimus, intravenous immune globulins, anti-TNF-alpha (etanercept), anti-lymphocyte globulin, high-dose steroids, immune checkpoint inhibitors, CAR-T cell related encephalopathy syndrome (CRES) with Chimeric Antigen Related-T cell therapy Other medications: lindane, permethrin, flumazenil, 4-aminopyridine (dalfampridine), sulfasalazine, theophylline, anti-histamines, opiates (morphine, tramadol) **Complementary and alternative medicines:** Borage oil, neem oil **Environmental toxins:** Lead, aluminum star fruit (oxalic acid, caramboxin), organophosphates, organochlorines and pyrethroids **Biotoxins:** Scorpion toxin, anatoxin, ciguatoxin, domoic acid and cyanide **Substances:** Benzodiazepines, amphetamines, cocaine, fentanyl, alcohol, ecstasy, heavy metals, synthetic cannabinoids, bath salts, LSD, heroin, PCP, marijuana **Consider:** Extended opiate and overdose panel
*Section 6: Neurologic exam*
Acute lower motor neuron syndrome: Japanese Encephalitis virus, West Nile virus, Tick-borne virus, Enterovirus (serotype 71, coxsackie) Acute parkinsonism: Japanese virus, Saint-Louis virus, West Nile virus, Nipah virus, *T. Gondii* Prominent oro-lingual dyskinesias, catatonia, neuropsychiatric and autonomic dysfunction: anti-NMDA receptor encephalitis Facio-brachial dystonic seizures, piloerection, paroxysmal dizzy spells and hyponatremia: anti-LGI-1 encephalitis Stiff person syndrome, hyperekplexia: anti-GAD 65 Mood changes and movement disorder: anti-mGLU-R Sensory neuronopathy/autonomic dysfunction: ANNA-1/anti-Hu Stiff person syndrome, progressive encephalomyelitis with rigidity and myoclonus, transverse myelitis: anti-amphiphysin antibody, anti-glycine Ataxia—Epstein-Barr virus, mitochondrial disorder
*Section 7: EEG findings*
Extreme delta brush: anti-NMDA receptor encephalitis Frontal-central slow wave contralateral to tonic-dystonic seizures: anti-LGI1 encephalitis Extreme spindles: *M. pneumoniae* Parieto-occipital epileptiform discharges and seizures: mitochondrial disorder including POLG1, PRES
*Section 8: MRI findings*
Prominent mesial temporal lobe involvement: paraneoplastic and autoimmune limbic encephalitis, anti-VGKC complex encephalitis (e.g., anti-LGI-1, anti-CASPR2) Basal ganglia: Saint-Louis encephalitis virus, La Crosse virus, Murray Valley virus, acute necrotizing encephalopathy of childhood (RANBP2 mutation) Posterior reversible encephalopathy syndrome (PRES) images: symmetrical cortical and subcortical hyperintense signals on T2 and FLAIR-weighted images in the parieto-occipital lobes of both hemispheres Stroke-like images: POLG1, MELAS
*Section 9: Auto-immune/paraneoplastic*
**Serum and CSF paraneoplastic and autoimmune epilepsy antibody panel:** Antibodies to LGI-1, CASPR2, Ma2/Ta, DPPX, GAD65, NMDA, AMPA, GABA-B, GABA-A, glycine receptor, anti-Tr, amphiphysin, CV-2/CRMP-5, Neurexin-3alpha, adenylate kinase, anti-neuronal nuclear antibody types 1/2/3 (Hu, Yo and Ri), Purkinje cell cytoplasmic antibody types 1,2, GFAP-alpha, anti-SOX1, N-type calcium channel Ab, PQ-type calcium channel **Other serologies:** ANA, ANCA, anti-thyroid antibodies, anti-TG anti-dsDNA, ESR, CRP, ENA, SPEP, IFE, antibodies to Jo-1, Ro, La, Scl-70, RA factor, ACE, anti-endomysium antibodies, cold and warm agglutinins Optional: consider storing extra frozen CSF and serum for possible further autoimmune testing in a research lab **Neoplastic:** CT chest/abdomen/pelvis, scrotal ultrasound, mammogram, pelvic MRI, CSF cytology and flow cytometry Optional: bone marrow biopsy; whole body PET-CT; cancer serum markers.
*Section 10: Metabolic/genetic*
**Metabolic:** See section 1 Ammonia, porphyria screen (spot urine), plasma and CSF lactate and pyruvate Consider: Vitamin B1 level, B12 level, pyridoxine, folate, CPK, troponin; tests for mitochondrial disorder (lactate, pyruvate, MR spectroscopy, muscle biopsy), tests for MAS/HLH (serum triglycerides and sIL2-r) **Genetic:** Screens for MERRF, MELAS, POLG1 and VLCFA screen. Consider ceruloplasmin and 24-h urine copper Consider whole exome or whole genome sequencing (also look for gene polymorphisms in IL1B, IL6, IL10, TNFA, IL1RN, SCN1A and SCN2A), mitochondrial genome sequencing, CGH array and genetics consult
*Section 11: Cytokine assay*
Cytokine assay for quantitative measure of-IL-1β, IL-1Ra, IL-4, IL-6, IL-8, IL-10, IL-12p70, IL-17A, CCL2/MCP-1, CCL3/MIP-1α, granulocyte colony stimulating factor (G-CSF), vascular endothelial growth factor (VEGF) and tumor necrosis factor-α (TNF-α), interferon gamma IFN-g Consider repeating the analyses during SE course

### Additional CSF testing

CSF cytokines may serve as markers of disease progression and may help choose treatment (8, 9, 27). A strong suggestion towards the involvement of innate immunity in the pathogenesis of FIRES was shown in a prospective case-control study of FIRES in children that showed a selective upregulation of proinflammatory cytokines (IL-6) and chemokines (IL-8/CXCL-10) in FIRES when compared against the control groups of inflammatory and non-inflammatory CNS disorders (11). In contrast, most T-cell-associated cytokines (IL-2, IL-17A, etc.) and homoeostatic chemokines (CCL21, CXCL12, etc.) remained unchanged or were downregulated.

Another study showed Th1-associated cytokines and chemokines to be elevated in FIRES compared to a broader network of cytokine and chemokine elevation in encephalitis (28).

In a single patient, elevated CSF proinflammatory cytokines (IL-8 and IL-6) before treatment normalized after anakinra when seizure control was obtained as well (29). Although CSF and serum levels of endogenous IL-1R antagonist are elevated in FIRES, a functional deficiency likely fails to block the IL-1R signaling as reported in the CSF of this single patient. Anakinra treatment can overcome this deficiency as post-treatment CSF showed a robust suppression of IL-1R signaling in response to IL1β (12). Other studies have shown seizure termination after administering IL-1 antagonists (such as anakinra) and IL-6 blockers, such as tocilizumab (13), in patients with NORSE. Thus, although no randomized trials or other definitive studies have been performed, CSF cytokine assay should be considered in all patients to help assess and characterize neuroinflammation, follow disease severity and progression, and guide the selection of targeted immunotherapies (section 11, Table 1).

Metagenomic next-generation sequencing is a comprehensive evaluation of microbial and host genetic material (DNA/RNA) in the CSF that aims to identify the presence of any non-human genetic material (i.e., infectious agents). This has largely been used in research, mostly related to encephalitis, and is now available for clinical use (14). Whenever possible, extra CSF should be stored for future autoimmune antibody testing, cytokine assay, and metagenomic analysis.

### Imaging

MRI brain with gadolinium should be performed in all patients without contraindications within 48 h of presentation (8). Additional testing with MR or CT venogram/angiography should be performed if there is a suspicion of vascular malformations, cerebral venous sinus thrombosis, reversible cerebral vasoconstriction syndrome, CNS vasculitis, etc. Prominent mesial temporal lobe involvement may be seen in autoimmune and paraneoplastic limbic encephalitis, such as anti-VGKC complex encephalitis (15). Prominent basal ganglia involvement may be seen in viral encephalitides such as La-Crosse virus encephalitis (see section 8, Table 1 for others), but can also occur with anti-LGI-1 encephalitis, acute necrotizing encephalopathy of childhood (related to mutations in RANBP2), and other conditions (16–18). Stroke-like images may be seen in POLG1-related CNS disease and other mitochondrial disorders such as MELAS. All of those conditions (and many others) can present as NORSE.

Repeat MRI later during the hospitalization and in outpatient follow-up should be considered in all patients. This helps monitor disease evolution for any new MRI changes, which may help indicate the etiology and/or aid in prognostication. A higher proportion of patients will show abnormalities on follow-up imaging than the initial imaging, as shown in a retrospective FIRES study where the follow-up brain MRIs were abnormal in 87% of studies; in contrast, initial MRI showed abnormalities in 38% of patients (30). Repeat MRI also helps in assessing long-term changes due to the underlying disease or as a result of prolonged seizures. Progressive brain atrophy was seen in all 19 patients with super-refractory status epilepticus in a prospective study, where the degree of atrophy correlated with the SE duration but did not correlate with functional outcomes (31). Repeat MRI may help in assessing disease prognosis as well. Higher grades of periventricular white matter changes, leptomeningeal enhancement on the initial MRI, and hippocampal atrophy on later MRIs predicted poor functional outcomes in one large series, as did extra-temporal lesion extension, including the claustrum (31–33). A 13-patient series of NORSE with limbic encephalitis from Korea observed that on follow-up imaging, 10/13 had extra-temporal lesion extension, most commonly to the claustrum (32). This was seen in all patients in another 31-patient series from Italy, about ten days after SE onset (often not present on the first scan), in the form of T2/FLAIR hyperintensity in bilateral claustra (34). While this sign was initially thought to be a part of the imaging changes related to prolonged ictal activity due to its observation in unusually severe cases of refractory status epilepticus, further studies are needed to clarify this. The two studies described here have shown a higher prevalence than any previous series. All patients in the study from Korea and 50% of those in the study from Italy had evidence of limbic encephalitis; autoimmunity has been proposed as a likely mechanism and a reason for the high prevalence of claustrum involvement in these studies. In our experience, the claustrum sign appears to be much less common in North America, but this warrants further investigation, regardless.

Magnetic resonance spectroscopy (MRS) should be considered in cases where inborn errors of metabolism (including mitochondrial disease) are suspected. Malignancy screening (CT of the chest, pelvis, and abdomen) should be performed in most or all patients with cryptogenic NORSE/FIRES, especially in adults. If negative, this should be followed by a testicular/ovarian ultrasound. Malignancy screening should include whole-body positron emission tomography (PET) when other testing remains negative, especially (but not only) in older adults (Figure 1) (8, 9).

### Continuous EEG

Continuous EEG monitoring is necessary for the diagnosis of non-convulsive seizures and for monitoring treatment effects with various medications. Certain EEG findings may point to the etiology, such as extreme delta brush in autoimmune encephalitides, particularly anti-NMDA receptor encephalitis (35), extreme spindles in *Mycoplasma pneumoniae* related infections (36), and parieto-occipital location of seizures and discharges in mitochondrial diseases (37). Tonic-dystonic seizures preceded by a contralateral frontal-central slow wave (∼580 ms and amplitude ∼71 μV) on EEG are seen in anti-LGI-1 encephalitis (19, 20).

### Genetic testing

The multinational expert panel agreed that genetic testing, including mitochondrial gene testing and neuroinflammation panel (38), should be considered early in young children and should be considered at some point in most patients with cryptogenic NORSE/FIRES. This may be followed by whole exome sequencing (8, 9), as several rare genetic and mitochondrial disorders can cause status epilepticus. Mitochondrial disorders associated with mutations of the genes encoding the presynaptic dynamin 1-like protein (DNM1L) and the catalytic subunit of mitochondrial DNA polymerase gamma (POLG1) have been seen in NORSE (39–44). Mutations of genes encoding neuronal channels such as *SCN1A*, *SCN2A*, and *SCN10A* have also been associated with NORSE (45–47). However, despite phenotypic similarities with certain genetic epilepsies, extensive genetic evaluation for candidate genes *PCDH19*, *SCN1A*, and *POLG* mutations was unrevealing in a cohort of pediatric FIRES patients (48). Another study of exome sequencing in 50 individuals (29 patient-parent trios and 23 single probands) with pediatric FIRES showed no pathogenic variants in genes associated with epilepsy or neurodevelopmental disorders; HLA sequencing in 29 patients did not show any allelic associations when compared against 529 population controls (49).

### Brain biopsy

Brain biopsy should be considered when a targetable lesion is identified by neuroimaging (and is not likely to be secondary to seizure activity) and avoided if there is no targetable lesion (8, 9). Neuropathology has been reported only rarely; when diagnostic, the findings have shown herpes simplex encephalitis, candida encephalitis, acute disseminated encephalitis vasculitis, necrotizing vasculopathy, and lymphocytic infiltration related to anti-GAD antibody disease (50, 51).

Only 15/197 (7.6%) patients were reported to have undergone a brain biopsy in a recent systematic review of NORSE/FIRES. In a series of 22 children with FIRES, only a third had a brain biopsy, and these revealed non-specific findings (52). In the absence of radiological lesions to target, the diagnostic yield of a brain biopsy was thought to be low by the expert panel. When brain biopsy is performed, metagenomic next-generation sequencing should be considered on the tissue for infectious disease evaluation, including for rare, unsuspected organisms.

## Treatment approach

Attempts at controlling status epilepticus should run parallel with disease modification efforts of the presumed disease, even when the etiology is unknown. Acute treatment of seizures should be similar to treatment of RSE in any situation. However, in patients without a clear explanation for SE in the first day or two, one should strongly consider first-line immunotherapy in the form of steroids, IVIG, or plasmapheresis; the consensus recommendations are to start these within the first 72 h of the onset of RSE (8, 9).

### Seizure suppression

#### Anti-seizure medications

The initial management of status epilepticus should be guided by local/institutional guidelines or published guidelines (53, 54). For convulsive status epilepticus, benzodiazepines are the first-line treatment. Levetiracetam, valproate, and fosphenytoin were equally efficacious as the second-line ASM for convulsive status epilepticus in the ESETT trial (55). If there is a concern for mitochondrial disorders, valproate should be avoided. Other ASMs available in an intravenous form for rapid administration, which are often appropriate for early use, include lacosamide, phenobarbital, and brivaracetam. If the parenteral medications fail to control the seizures, enteral medications can also be tried (via a nasogastric tube). Continuous EEG monitoring is required to manage these patients, even those beginning as convulsive SE, as the seizures virtually always become nonconvulsive. The medications that do not show efficacy should be discontinued to avoid the accumulation of ASM burden with the potential side effects from the polypharmacy. There is no data to suggest what specific anti-seizure medications or a combination might be effective in this setting. However, published expert consensus suggests treating seizures in patients with NORSE/FIRES the same as with other causes (8).

#### Anesthetics

Anesthetic drug use should be similar to treatment of RSE in other conditions during the initial 48 h of NORSE/FIRES management (8, 9). Current data do not support using one anesthetic agent over any other. The commonly used anesthetics are midazolam, propofol, pentobarbital, thiopental, and ketamine. High-risk patients should be monitored to avoid and treat propofol infusion syndrome (56). Propofol, pentobarbital, and thiopental should be used with caution in mitochondrial disorders due to possible association with hepatic dysfunction (42). Limited data have shown favorable hemodynamics with ketamine, or at least less hypotension than with other anesthetics (57). Pentobarbital or thiopental is usually considered after other anesthetics fail, as they are associated with hypotension, electrolyte abnormalities, infections, and ileus.

The neurocritical care society guideline for evaluating and managing status epilepticus discusses the dosing considerations for the above-described anesthetic agents (58). There are no high-quality data to support the intensity and duration of anesthetic agents. The titration of the anesthetic agent is guided by continuous EEG, with the goal being the suppression of seizures or a background pattern of burst suppression. Titration to suppression-burst was associated with a lower frequency of seizure recurrence than titration to suppression of seizures; however, it was also associated with a significantly higher frequency of hypotension in a meta-analysis (59) Neither the choice of the anesthetic agent nor the titration goal was associated with differences in the overall outcome. The guidelines recommend seizure control for 24–48 h before a gradual taper of the anesthetics with ASMs in place for maintenance; recurrence of seizures post-anesthetic wean warrants resumption of anesthesia, likely at a higher dose (58). While the usual goal is to suppress most or all seizures, if aiming for suppression burst, experts recommend an interburst interval of 10 s and to wean anesthetic over 6–12 h. A recent retrospective study of propofol used for RSE showed that a shorter trial at higher doses might be more effective and safer than the recommended therapeutic coma duration (60). In this study, the duration of an initial therapeutic coma longer than 35 h was associated with a higher risk of seizure recurrence following the anesthetic wean. These findings align with a previous retrospective study of midazolam in RSE that showed lower mortality for a higher dose of midazolam (2.9 mg/kg/h) than a lower dose (0.4 mg/kg/h) (61). However, earlier retrospective studies have found therapeutic coma to be associated with poor outcomes, but the confounding effect of the refractoriness of SE (that required anesthetic use) could explain the poor outcomes. Therapeutic coma in the setting of focal status epilepticus, especially with fully or partially maintained awareness, has been argued against due to similar concerns shown in another study that looked at outcomes in generalized vs. focal status epilepticus (62). Expert recommendations favor managing focal status epilepticus without significant impairment of consciousness without anesthetics (63).

#### Ketogenic diet

The expert panel recommends starting the ketogenic diet in the first week of hospitalization in children still in RSE. It should be considered in all prolonged and severe cases, including in adults, if not already given in the first week. If enteral administration is not possible, parenteral administration should be considered (if expertise is available for guidance) (8, 9). The ketogenic diet was shown to effectively control seizures within a few days of ketonuria in a pediatric FIRES series (64, 65) and was the only therapeutic agent that possibly shortened the acute phase in a retrospective study of 77 children with NORSE (66). Retrospective studies of refractory and super-refractory status epilepticus in adults and children have shown the ketogenic diet to be effective with only mild side effects (67, 68). The feasibility of a ketogenic diet in adults with RSE in the intensive care setting has been shown in some reports; however, the institutional expertise may vary even at tertiary care centers (69, 70).

### Disease modification

Steroids are the first-line immunologic agent and should be started within 72 h of admission, preferably earlier if the initial etiologic workup is complete. Methylprednisolone 20–30 mg/kg per day (max 1 gm) should be given for 3–5 days intravenously. Intravenous immunoglobulins can be an alternative to steroids (see Figure 1 for doses) or can be administered simultaneously with steroids (9). The response to first-line immunotherapy is often incomplete. Once infections are excluded, second-line immunologic treatment should start within seven days of the onset of RSE, but it has the potential to improve outcomes even if administered after several weeks. Rituximab is recommended if an antibody-mediated disease is suspected or confirmed. In cryptogenic NORSE, IL-1R antagonists or IL-6 antagonists should be used, at least based on the current (limited) state of knowledge (8, 9). There is not high-quality data supporting second-line immunotherapy use other than anecdotal experience. Additionally, the results from case reports and series should be interpreted cautiously due to the confounders of publication bias and a natural disease course. The expert panel recommendation is based on experience with these agents in other neuroinflammatory disorders and risk-benefit assessment. The evidence does not support using a specific agent for second-line immunotherapy.

Anakinra is a recombinant interleukin-1 receptor antagonist used to treat rheumatoid arthritis, Still’s disease, and cryopyrin-associated periodic syndromes. Multiple case reports have shown the benefit of anakinra in NORSE/FIRES patients that fail first-line and second-line immunotherapy (71–73). A retrospective study of 25 children treated with anakinra for FIRES showed association of treatment with shorter duration of mechanical ventilation, ICU and hospital length of stay. One treatment discontinuation was noted due to infection (74). Anakinra has also been used in other CNS inflammatory disorders. Four out of twelve adult patients receiving anakinra for various cerebral autoinflammatory disorders (including primary progressive multiple sclerosis, ADEM, autoimmune encephalitis, NORSE) etc. showed good outcomes following treatment, and none had any serious adverse events (75, 76). Tocilizumab is a humanized monoclonal antibody against the IL-6 receptor, which has been used in rheumatoid arthritis, giant cell vasculitis, and cytokine release syndrome. In a 7-patient series of NORSE, treatment with tocilizumab was effective for 6/7 patients that failed conventional immunotherapy, including rituximab (13). One was attributed to NMDA antibodies, but the rest were cryptogenic. All patients had a prolonged course ranging from 16–75 days and failed multiple drugs and three anesthetic agents. Adverse events included severe infections in 2 and leukopenia in 3. Outcomes were not significantly different from other series, but the authors argue that this series was likely biased by including prolonged and severe cases; earlier administration of tocilizumab may have the potential for better outcomes. In cryptogenic cases of NORSE, failure of benefit with anakinra does not preclude a trial of tocilizumab, and vice-versa (77, 78).

In a series of the chronic phase of FIRES, anakinra was effective in 3/5 patients with a significant reduction in seizure burden without additional serious adverse effects. One patient had to be switched to tocilizumab due to inefficacy. This was studied against a control group that included nine patients, and only one had mild improvement in seizure frequency in a 6-month follow-up period (78–80). Randomized controlled studies are necessary to shed further light on the efficacy of disease-modifying treatment but are challenged by the rarity of this condition.

### Other treatments: neuromodulation/cannabidiol/hypothermia/intrathecal steroids

Non-invasive and invasive neuromodulation methods are feasible as a treatment option for super-refractory status epilepticus, including NORSE. Transcranial magnetic stimulation, electroconvulsive therapy, vagus nerve stimulation, and deep brain stimulation have all been sporadically used to manage super-refractory status epilepticus with variable benefits (80).

Similarly, there are isolated case reports of positive results with responsive neurostimulation with and without focal brain resection for super-refractory status epilepticus and NORSE/FIRES (81, 82). Current evidence does not support cannabidiol or hypothermia as a first or second-line treatment (8, 9). Functional outcomes were no different between the hypothermia and control groups in a randomized control trial for convulsive SE (83, 84). It has been reported to be effective in a few cases, but the level of evidence is likely inadequate to justify the risks at the current time (85, 86). Lastly, intrathecal steroids have been used: In a study of six children with FIRES, a shorter time from disease onset to treatment with intrathecal dexamethasone correlated with a shorter ICU stay and mechanical ventilation with no serious adverse events (87). Intrathecal steroids have the potential to shorten the acute stage of the disease, but further studies are needed.

### Palliative care

NORSE is a heterogeneous condition whose etiology remains unidentified in many patients leaving the prognosis uncertain. Physicians should keep open communication with family regarding prognosis and the uncertainty involved. The palliative care team can effectively facilitate these conversations with the family and many other aspects of care and should be involved early. It is important to recognize that consulting palliative care does not mean that aggressive treatment is being abandoned; i.e., palliative care is not equivalent to hospice care. Due to the involvement of multiple specialists over a long period of time, identifying a lead physician that integrates all the data to present to the family is desirable (88, 89).

### Discharge planning

Most patients will benefit from intensive motor and cognitive rehabilitation before a home disposition. Many will need ongoing immunologic treatments and multiple anti-seizure medications (ASMs) to manage pharmacoresistant epilepsy. Some patients (those with cryptogenic NORSE) will benefit from an ongoing evaluation with repeat imaging, including brain MRI and consideration of repeat imaging for malignancy screen. In those with poor seizure control, surgical evaluation for epilepsy surgery should be considered (Figure 1).

### Outcomes/chronic disease

Mortality during the acute phase is seen in 13%–30% of adults and children (40, 90). Of the survivors, about two-thirds develop epilepsy (higher in pediatric series and lower in adults), with about half being drug resistant (40, 91). Poor functional outcomes are seen in about two-thirds of survivors. Studies of children with FIRES have shown that functional outcomes improve in most patients over time, with good outcomes (though usually not return to baseline) seen in two-thirds of the survivors at the last follow-up. A good outcome has been reported despite a prolonged therapeutic coma lasting for several months during the acute hospitalization (92).

Most patients need anti-seizure medications at discharge. If no clinical seizures are seen for three months following discharge, medication taper can be attempted with the goal of discontinuation. Prolonged EEG (24–72 h), usually performed in the outpatient setting (ambulatory EEG), can guide medication taper. For patients with continued drug-resistant epilepsy, immunotherapy (rituximab/anakinra/tocilizumab) should be continued at discharge, with a re-assessment of the need at three months. Patients who continue to have seizures despite use of ASMs, with or without immunotherapy, should be evaluated for epilepsy surgery, including neuromodulation.

## Conclusion

NORSE, including its subtype of FIRES, is a rare and often devastating condition that presents with refractory and often super-refractory status epilepticus. The etiology is heterogeneous, with no definite one found in the majority of cases, but inflammation with activation of innate immunity is likely an important component of the pathophysiology in many cases, especially the cryptogenic ones. Early treatment with first-line immunotherapy and timely introduction of the ketogenic diet and IL-1R/IL-6 antagonists should be considered in most super-refractory patients. The current evidence to support these treatments is limited, but several multinational research efforts are ongoing to help elucidate the pathogenesis and to study treatment options systematically. One such collaboration resulted in the creation of the NORSE Institute and an active biobank that is collecting and analyzing samples from patients with NORSE/FIRES around the world.^1^ The same website provides frequently-updated resources for clinicians, researchers, patients and families.

## Author contributions

ZS conceptualized the manuscript framework, did a literature review, drafted the manuscript, created the figure, and edited the tables. LH edited and revised the manuscript and contributed to the figure and tables. All authors contributed to the article and approved the submitted version.

## Conflict of interest

ZS is a member of the medical and scientific advisory board of the NORSE institute. LH has received consultation fees for advising from Accure, Aquestive, Ceribell, Eisai, Marinus, Medtronic, Neurelis, Neuropace, and UCB; royalties from Wolters-Kluwer for authoring chapters for UpToDate–Neurology and from Wiley for coauthoring the book Atlas of EEG in Critical Care by Hirsch and Brenner; and honoraria for speaking from Neuropace, Natus, and UCB. He serves as the co-chair of the medical and scientific advisory board of the NORSE institute.

## Publisher’s note

All claims expressed in this article are solely those of the authors and do not necessarily represent those of their affiliated organizations, or those of the publisher, the editors and the reviewers. Any product that may be evaluated in this article, or claim that may be made by its manufacturer, is not guaranteed or endorsed by the publisher.

## Footnotes

^1^ https://www.norseinstitute.org/
