# Corrigendum: A practical approach to in-hospital management of new-onset refractory status epilepticus/febrile infection related epilepsy syndrome

**DOI:** 10.3389/fneur.2024.1509148

**Published:** 2024-11-11

**Authors:** Zubeda Sheikh, Lawrence J. Hirsch

**Affiliations:** ^1^Department of Neurology, West Virginia University School of Medicine, Morgantown, WV, United States; ^2^Epilepsy Division, Department of Neurology, Yale School of Medicine, New Haven, CT, United States

**Keywords:** new-onset refractory status epilepticus, febrile infection related epilepsy syndrome, anakinra, tocilizumab, rituximab, super-refractory status epilepticus, neuroinflammation, autoimmune encephalitis

In the published article, there was an error in Figure 1 as published. The dose of IVIG was listed as 2 mg/kg over 2–5 days, when it should be 2 g/kg over 2–5 days.

**Figure 1:**
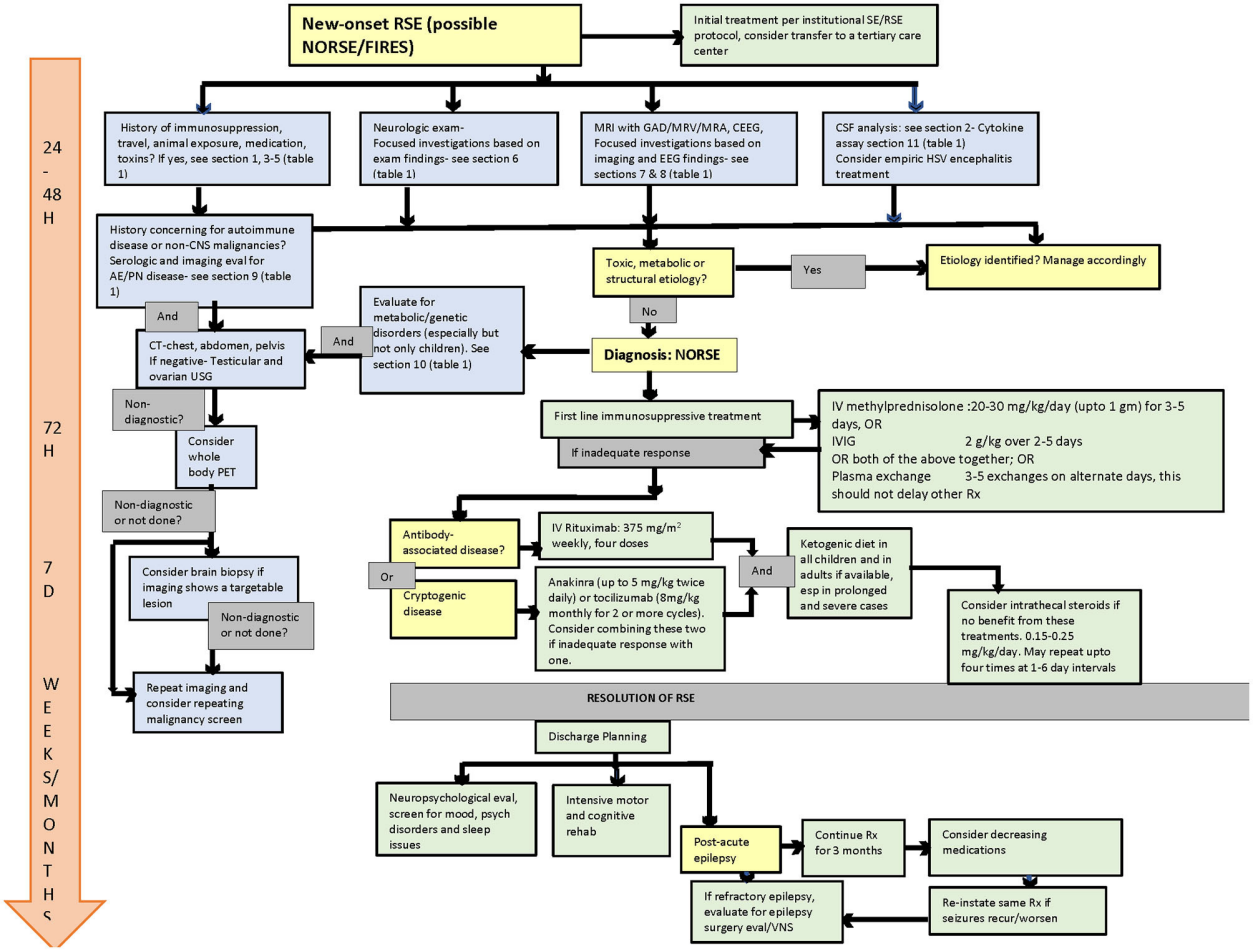
Flow diagram for evaluation and management of NORSE/FIRES. NORSE, New Onset Refractory Status Epilepticus; FIRES, Febrile infection-related epilepsy syndrome; SE, Status epilepticus; RSE, Refractory status epilepticus; GAD, Gadolinium; MRV, Magnetic resonance venogram; MRA, Magnetic resonance angiography; CEEG, Continuous EEG; USG, Ultrasonography; PET, Positron emission tomography; HSV, Herpes simplex virus; VNS, Vagus nerve stimulator. 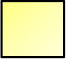
Diagnostic consideration. 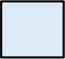
Diagnostic procedure. 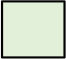
Treatment. 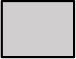
Outcome assessment. 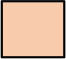
Management timeline.

The corrected Figure 1 and its caption appear below.

The authors apologize for this error and state that this does not change the scientific conclusions of the article in any way. The original article has been updated.

